# Editorial: Ethanol, Its Active Metabolites, and Their Mechanisms of Action: Neurophysiological and Behavioral Effects

**DOI:** 10.3389/fnbeh.2018.00095

**Published:** 2018-05-17

**Authors:** Elio Acquas, John D. Salamone, Mercè Correa

**Affiliations:** ^1^Department of Life and Environmental Sciences, University of Cagliari, Cagliari, Italy; ^2^Department of Psychology, University of Connecticut, Storrs, CT, United States; ^3^Department of Psychobiology, Universitat Jaume I, Castelló, Spain

**Keywords:** acetaldehyde, adenosine, caffeine, D-penicillamine, ethanol metabolism, fenofibrate, nicotine, salsolinol

Over the last century the neurobiology of ethanol has come a long way since the original proposal that the two main pharmacological effects, intoxication and sedation, could be explained by the ability of ethanol to cause perturbation of neuronal membrane lipids (Meyer, 1901). This view, although questioned on the basis of the advancement of knowledge on the effects of ethanol on membrane proteins and intracellular kinases (Tabakoff and Hoffman, 2013), continued to be highly considered up to the late'80s, whereby disruption of the order or increase of the fluidity of biological membranes was still identified as a critical determinant for its biological and behavioral effects (Kalant, 1975).

Current views on the neurobiology of ethanol have been based on preclinical studies conducted in the last few decades. New approaches are based on processes ranging from identifying ethanol-sensitive molecules to determining the role of such molecules in ethanol-mediated physiological and behavioral changes (bottom-up), but also by establishing the correlation between ethanol-dependent physiological and behavioral effects and the involvement of specific molecular mechanisms (top-down). These views presently allow an in-depth distinction between direct (ion channels, protein kinases) and indirect (intracellular signaling proteins, growth and transcription factors) molecular targets (Abrahao et al., 2017). In addition, by virtue of its peripheral and central metabolism, ethanol generates a number of biologically active molecules. This raises the need for characterization of the relationship between ethanol, its metabolites (mainly acetaldehyde but also acetate and salsolinol), and some of their central and peripheral effects. In this regard, acetaldehyde in particular has received a great deal of attention. After the serendipitous observation made by Chevens (1953) that patients under treatment with the aldehyde dehydrogenase (ALDH) inhibitor antabuse experienced pleasurable effects upon taking small amounts of ethanol, and the discovery of catalase-mediated central metabolism of ethanol (Aragon and Amit, 1985), it was recognized that acetaldehyde could be significantly mediating ethanol's stimulant effects on behavior. These discoveries led to the recognition of acetaldehyde as a pharmacologically neuroactive molecule (Correa et al., 2012). Along the way there have been long discussions of controversial issues such as the uncertainties surrounding central acetaldehyde determination (discussed in the present topic by the review of Enrico and Diana), and the ability of acetaldehyde to cross the blood brain barrier. More recently, much attention has focused on the differential role of acetaldehyde in acquiring and maintaining voluntary ethanol intake (thoroughly discussed in the contributions by Israel et al. and Peana et al.).

In view of the continued progress being made in research on ethanol and its metabolites, we decided to host this research topic 4 years after another successful research topic “Neuroactive metabolites of ethanol: a behavioral and neurochemical synopsis” (Correa et al., 2014). We reasoned that widening the perspective beyond ethanol's active metabolites to ethanol's neurophysiological and behavioral effects, as well as proposed mechanisms of action, would result in an up-to-date and integrated view of current research on the neurophysiological and behavioral effects of ethanol and its active metabolites.

The four review papers of this topic make a picture of the *state of the art* of preclinical research on the role of ethanol metabolites, ethanol intake, and ethanol-elicited motivated behavior. Israel et al. discuss the recent evidence gained using high-ethanol drinker rats of the UChB line, pointing to the differential role played by acetaldehyde in acquisition, maintenance, and relapse. In particular, whereas acetaldehyde availability seems critical for the initial boost (first hit) of ethanol self-administration (acquisition), and also for relapse after long term withdrawal, this does not apply to the maintenance phase since that is not prevented by the interference with catalase-mediated central metabolism of ethanol. A similar view is presented in the paper by Peana et al. which also discusses the recent hypothesis that 4-methylpyrazole may indirectly affect central as well as peripheral metabolism of ethanol. In fact, mainly known for its ability to inhibit alcohol dehydrogenase (ADH), 4-methylpyrazole may also affect the availability of hydrogen peroxide to act as a factor critical for catalase-mediated oxidation of ethanol. In addition, the reviews by Israel et al., and Peana et al. place a great emphasis on salsolinol as a bioactive molecule that shares locomotor stimulant and motivational properties with ethanol and acetaldehyde. In this regard, the paper by Berrios-Carcamo et al. adding to the recent demonstration that systemic administration of salsolinol exerts central effects such as behavioral sensitization and conditioned place preference, provides fresh and elegant biochemical (classical G protein-adenylate cyclase pathway assessments), and molecular (docking simulations using the crystal structure of the mouse μ opioid receptor) evidence in support of the suggestion that the central actions of salsolinol are mediated by μ opioid receptors.

A distinct and original point of view in regards to the ethanol-acetaldehyde relationship is provided by the contribution of Brancato et al. Besides focusing on the acetaldehyde's mirroring effects of ethanol in the brain, these authors center the discussion of their paper on the relevance of three critical players (a “ménage à trois” on their words) worth of further investigations: (1) the mesolimbic dopamine system, (2) the stress response system, due to the acetaldehyde-mediated activation of the hypothalamic-pituitary-adrenal (HPA) axis and increased CRH and NPY expression, and (3) the endocannabinoid system, due to the ability of CB1 genetic deletion and receptor antagonists to prevent behavioral and neuroendocrine effects of acetaldehyde.

In another review Virgolini et al. discuss the available literature on the ability of the environmental contaminant lead (Pb) to affect the motivational properties of ethanol. This analysis, based on the observation that Pb may affect catalase-mediated central ethanol metabolism as well as ethanol intake, highlights that early exposure to Pb may increase susceptibility to engage in abnormal ethanol taking behaviors through an interference with its central metabolizing enzymes.

Two contributions to this research topic focus on the role that pre-natal exposure to acetaldehyde may have on post-natal acceptance to ethanol as well as on respiratory plasticity in newborns. Gaztañaga et al. report that acetaldehyde acts as a reinforcer in the appetitive learning that occurs upon ethanol exposure during the late gestational days. In particular, this paper shows that pre-natal brain acetaldehyde formation via catalase may be responsible for post-natal acceptance of ethanol, evidence gained by studies involving administration of ethanol and the acetaldehyde-sequestering agent, D-penicillamine, to dams. Moreover, an evaluation of the consequences of exposure of the immature brain to ethanol and acetaldehyde in terms of subsequent self-administration procedures is offered by the study from Acevedo et al. demonstrating that early exposure to both compounds exerts similar effects on respiratory plasticity and thermoregulatory alterations of the neonates as well as on seeking behavior of ethanol as a reinforcer in an operant task in neonate rats.

The ability of previous exposure to ethanol or to environmental enrichment in modulating ethanol consumption in adulthood has been taken into account in the studies by Carrara-Nascimento et al. and Berardo et al. In particular, using a three-bottle choice paradigm to evaluate the escalation into ethanol consumption in adulthood, Carrara-Nascimento et al. showed that rats that received ethanol during adolescence had greater intake of ethanol. Berardo et al. on the other hand, analyzed, in male and female rats, the consequences of early-life exposure to maternal separation (post-natal days 1–21) and environmental enrichment (post-natal days 21–42) on ethanol consumption and found that male but not female rats exposed to environmental enrichment consume more ethanol during late adolescence in a two-bottle intake procedure than controls, a result not affected by previous experience of maternal separation. Moreover, since heightened exploration of novel stimuli and greater risk-taking behaviors were more evident in male rats exposed to enriched environments, these authors postulate that such increases in ethanol consumption could be due to the effects of exposure to enriched environment upon exploratory and risk-taking behaviors.

The suggestion of potential therapeutic approaches for preventing relapse in alcoholism and abnormal ethanol taking behaviors, which originated based on preclinical evidence, has been dealt with in the contributions by Orrico et al. for the suggestion of D-penicillamine, and by Rivera-Meza et al. for the suggestion of the peroxisome proliferator-activated receptor alpha (PPARα) agonist, fenofibrate. In particular, Orrico et al. discuss recent evidence on the effectiveness of the acetaldehyde sequestering agents in the alcohol deprivation effect, a reliable operant rodent model of relapse-like drinking behavior, which allowed a comparison of the effectiveness of D-Penicillamine with other FDA approved medications such as Acamprosate, Nalmefene and Naltexone. However, based on the conflicting evidence that D-penicillamine may or may not represent a valid pharmacological approach against voluntary ethanol intake in long-term experienced patients, the authors conclude that their suggestion of D-penicillamine as a therapeutic agent against relapse necessitates full clinical testing either alone and in association with other FDA approved medications such as nalmefene.

Using high drinkers UChB rats Rivera-Meza et al. extend their own previous work on the ability of fenofibrate to affect voluntary ethanol intake by a peripheral action linked to increased liver catalase expression and, hence, to increased peripheral acetaldehyde, to the possibility that fenofibrate may also act by a centrally-mediated mechanism. To address this point, the authors evaluated the ability of fenofibrate to affect ethanol-elicited conditioned place preference and voluntary ethanol or saccharine intake. The results of the study show that fenofibrate prevents ethanol-elicited conditioned place preference but also decreases ethanol and saccharin intake, thus supporting the suggestion that its actions might be ascribed to both peripherally- and centrally-mediated mechanisms, perhaps linked to catalase overexpression in the liver but not in the brain.

The contribution by Bassareo et al. provides original evidence of the involvement of nucleus accumbens shell and core dopamine transmission in response-contingent 10% ethanol self-administration under a FR1 schedule of nose-poking, and compares this involvement with that of 20% sucrose and of 10% ethanol + 20% sucrose. The results of this study reveal that active ethanol self-administration similarly increases dopamine transmission in the shell and core subdivisions, whereas under extinction trial this is preferentially increased in the shell of the accumbens. In contrast, under sucrose operant taking and extinction, dopamine transmission increases selectively in the shell overall demonstrating that the 10% ethanol self-administration procedure, without the interference of moving the animals from the home cage to the operant box, increases dopamine in both accumbens subdivisions and that these play different roles in sucrose as compared to ethanol reinforcement stimuli.

Finally, two research papers address the point of how other highly consumed drugs (nicotine and caffeine) can interfere in ethanol's actions. Coming from the perspective that tobacco use presents a strong positive correlation with alcohol use, the interesting research by Lárraga et al. investigates the relationship between exposure to 10 days of nicotine, or ethanol, or nicotine + ethanol intravenous self-administration, age (adolescents or adults) and sex on ethanol intake in adulthood determined using the two-bottle choice procedure. The results of this longitudinal study indicate major age- and sex-dependent differences whereby adolescent males that appear more sensitive to the reinforcing effects of nicotine + ethanol also result to have greater ethanol intake, suggesting that early exposure to nicotine may determine greater vulnerability to alcohol abuse. Authors conclude, in a translational perspective, that this evidence provides strong support for the suggestion to limit adolescent access to nicotine and tobacco products (including e-cigarettes). The research by López-Cruz et al. focuses on the possible impact that ethanol, caffeine and their interaction may exert on motivation for social contact (recognition and memory) as assessed in CD-1 mice in a three-chambered box. Based on the observations that ethanol affects social interaction in a biphasic manner without affecting social preference, while caffeine reduces social contact and blocks social preference, and that ethanol and caffeine have opposite effects on adenosine system, this study tests the hypothesis that a common mechanism of action, via the adenosine system, may regulate these opposite actions. Results showed that ethanol, at appropriate doses, could reverse the caffeine-mediated reduction of social exploration. However, given that selective antagonists of the adenosine A_1_ and A_2A_ receptor subtypes do not mimic the effects of caffeine, the authors conclude, from a translational perspective, that the usefulness of highly caffeinated drinks in counteracting high doses of ethanol-induced impairments in social processes is questionable.

## Author contributions

All authors listed have made a substantial, direct and intellectual contribution to the work, and approved it for publication.

### Conflict of interest statement

The authors declare that the research was conducted in the absence of any commercial or financial relationships that could be construed as a potential conflict of interest.
